# A Unique Case of Systemic Lupus Erythematosus Pelvic Vasculitis

**DOI:** 10.1155/2016/6347901

**Published:** 2016-12-29

**Authors:** Pamela Traisak, Shristi Basnyat, Hala Eid, Patrick Cronin, Halyna Kuzyshyn, David Feinstein

**Affiliations:** Division of Rheumatology, Cooper University Hospital, Camden, NJ, USA

## Abstract

The clinical presentation of Systemic Lupus Erythematosus (SLE) is diverse and vasculitis can be a potential manifestation. Cutaneous lesions involving small vessels are the most frequent presentation. However, medium and large vessel vasculitis may present with life-threatening visceral manifestations. We present a unique case of pelvic vasculitis mimicking a pelvic mass as an initial presentation of SLE. There are case reports of systemic vasculitis involving the female genital tract with giant cell arteritis (GCA), polyarteritis nodosa (PAN), and granulomatous with polyangiitis and microscopic polyangiitis (GPA/MPA), among others, but only a few cases attributed to SLE. Awareness of this condition and a prompt diagnosis are warranted as this is a severe and potentially life-threatening condition.

## 1. Introduction

The clinical presentation of Systemic Lupus Erythematosus (SLE) is diverse and can potentially involve any organ or system. This variability is due to the production of an array of autoantibodies thought to cause tissue damage. Vasculitis prevalence in SLE is reported to be 11%–36% [1]. Involvement of small vessels with cutaneous lesions is seen most frequently. However, medium and large vessel vasculitis may present with life-threatening visceral manifestations. SLE involvement of the female genital system is extremely rare. We describe a case of pelvic vasculitis, mimicking a pelvic mass, as the initial manifestation of SLE.

## 2. Case Presentation

A 35-year-old African American female was admitted to an outside hospital with an 8-month history of abdominal discomfort, bloating, transient arthralgia, and unintentional weight loss of 60 pounds. A CT scan of the abdomen and pelvis showed a large complex left retroperitoneal mass, abdominal ascites, and pleural effusions, contiguous with the left adnexa. She was found to have hemoglobin of 4.9 g/dL and received blood transfusions prior to transfer to Cooper University Hospital.

Given the abnormal CT scan and her 60-pound weight loss, she underwent an exploratory laparotomy which revealed a large hematoma extending up the left pelvic wall to the kidney, mesosalpinx of the left adnexa, and 300 mL of ascites. As malignancy was a major concern, a total abdominal hysterectomy, right oophorectomy, bilateral salpingectomy, left pelvic lymph node dissection, omentectomy, and evacuation of the retroperitoneal hematoma were performed. Frozen tissue pathology was obtained and revealed hemorrhage and no malignancy.

**Figure 1:**
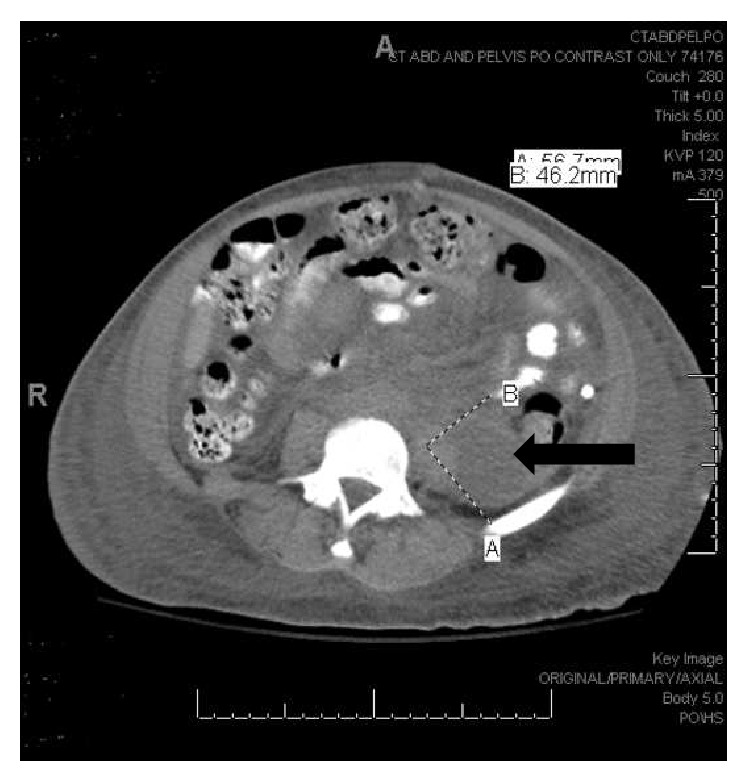
Repeat CT scan of the abdomen and pelvis with an ovoid fluid collection which measures 5.7 × 4.6 cm in the axial plane in the left posterior perinephric space.

After her surgery, the rheumatology service was consulted. Her exam was remarkable for a fever of 101.2 F, discoid lesions on her ears, a distended abdomen, and 1+ pedal edema. The abnormal labs included a hemoglobin of 8.2 g/dL (microcytic), lymphopenia 0.9 k/ul (with normal WBC = 6 k/ul), creatinine 1.4 mg/dL, and urinalysis with 3+ protein, 2+ blood, 16 WBCs, 36 RBCs, and 1+ granular casts. A repeat CT of the abdomen and pelvis (Figure 1) showed multiple pelvic and retroperitoneal fluid collections.

**Figure 2:**
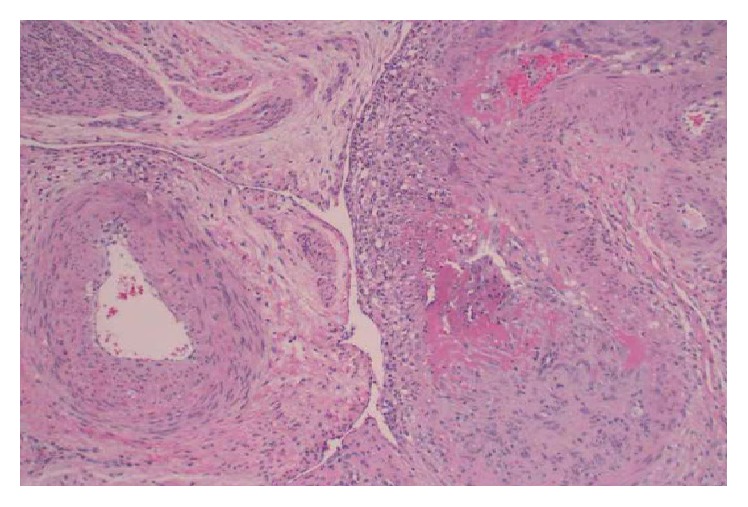
Vessels in the myometrium. Uninvolved vessel on the left: vasculitis involves vessels on the right. Normal myometrium upper left. Vasculitis characterized by an inflammatory infiltrate, mostly neutrophilic, involving vessel wall with fibrinoid necrosis and thrombus [H&E ×10].

Postoperatively, renal function worsened with associated oliguria. There was increased concern for an underlying glomerulonephropathy after records from the outside hospital revealed a positive ANA (1 : 1280), CH50 < 13, and 7 grams of proteinuria. Additional labs at our facility revealed positive ENA to SCL-70, anti-DNA, RNP antibody, Smith antibody, SSA antibody, and low complements. Antiglomerular basement membrane antibody, ANCA screen, rheumatoid factor, cryoglobulin, HIV-1/2 antibody, and hepatitis viral panel were all negative. The final pathology report revealed a left ovary/fallopian tube organizing hematoma, with vasculitis of the small and medium sized blood vessels involving the uterus, cervix, right fallopian tube, paratubal tissue, and omentum (Figure 2).

Due to the patient fulfilling both the 1997 ACR and 2012 SLICC SLE classification criteria, she received IV pulse methylprednisolone, followed by oral prednisone 1 mg/kg and mycophenolate mofetil. A kidney biopsy was deferred due to increased risk of hemorrhage. Her renal function continued to improve and she was discharged on prednisone 60 mg daily with immunosuppressive therapy.

## 3. Discussion

In the present case, a large retroperitoneal pelvic mass was unexpectedly discovered to be the result of a pelvic/uterine vasculitis associated with SLE. SLE pelvic/uterine vasculitis is a very unusual and rare entity. The patient's initial clinical presentation was suspicious for a gynecological malignancy. A diagnosis of SLE associated pelvic/uterine vasculitis was reached after patient was discovered to have proteinuria and additional evaluation for SLE was pursued.

Uterine involvement in SLE is rare and usually related to pregnancy. There are case reports of systemic vasculitis involving the female genital tract with GCA, PAN, GPA/MPA, rapidly progressive glomerulonephritis associated with perinuclear antineutrophil cytoplasmic antibody, and limited systemic sclerosis [2–5]. There are only a few cases attributed to SLE, after review of literature spanning nearly three decades.

Hernández-Rodríguez et al. reviewed characteristics of gynecologic vasculitis in an analysis of 163 patients and compared those with isolated gynecologic vasculitis (70.6%) to those with systemic vasculitis (29.4%) [6]. Compared to those with systemic disease, those with isolated presentation were younger, presented more often with vaginal bleeding (as opposed to a pelvic mass), and were less likely to have abnormal ESR or anemia. Among systemic vasculitis patients, giant cell arteritis was diagnosed in 60.4% of patients [6]. Only one patient was found to have vasculitis associated with SLE in this study and had renal involvement concomitantly.

Ramos-Casals et al. studied the prevalence and clinical characteristics of vasculitis in Systemic Lupus Erythematosus in 670 patients [7]. Seventy-six (11%) patients with SLE had vasculitis (68 female patients and 8 male; mean age, 37.8 yr.). Only 32 (42%) fulfilled the Chapel Hill definitions. Cutaneous lesions were the main clinical presentation of vasculitis, present in 68 (89%) patients, while the remaining 8 (11%) had isolated visceral vasculitis. Compared with SLE patients without vasculitis, patients with vasculitis had a higher prevalence of livedo reticularis (22% versus 3%), a higher mean European Consensus Lupus Activity Measurement (ECLAM) score (5.86 versus 3.87), a higher frequency of anemia (62% versus 17%), ESR > 50 mm/h (60% versus 15%), and anti-La/SS-B antibodies (19% versus 5%). Sixty-five (86%) patients had small vessel vasculitis (SVV) and 11 (14%) had medium sized vessel vasculitis (MVV). It was concluded that a heterogeneous presentation of vasculitides could occur in the setting of SLE, with nearly 60% of cases not fulfilling the definitions adopted by the Chapel Hill Consensus Conference [7].

Vasculitis is characterized by the presence of inflammatory cell infiltration into the vessel wall with subsequent necrosis. Vascular inflammation may occur by deposition of immune complexes. The presence of autoantibodies, such as antiendothelial cell antibody (in up to 80% of patients) and ANCA (in up to 20%), has been documented to be involved in the pathophysiology of lupus vasculitis [8, 9].

The primary basis of treatment for systemic vasculitis, in general, is suspected diagnosis and timely intervention. As with other vasculitides with involvement of visceral organs, the mainstay of treatment is corticosteroids and immunosuppressive therapy. Examples of immunosuppressive therapy include cyclophosphamide, mycophenolate mofetil, or azathioprine. Use of rituximab, belimumab, and IVIG has been described in refractory cases [1].

Specific to this case, pulse dose intravenous glucocorticoids were used due to the severity of patient's presentation and significant proteinuria with the suspicion for active lupus nephritis. Unfortunately, a kidney biopsy to establish the diagnosis of lupus nephritis and class was deferred due to a high risk for hemorrhage. Mycophenolate mofetil was chosen as the immunosuppressive therapy due to patient's African American race and preference over cyclophosphamide.

## 4. Conclusion

Pelvic/uterine vasculitis secondary to lupus remains a rare entity. However, awareness of this condition is important as it may present as the initial manifestation of SLE. A prompt diagnosis is warranted as this is a severe and potentially life-threatening condition.
